# A dual‐responsive fluorescent probe for imaging viscosity and polarity in nonalcoholic fatty liver disease

**DOI:** 10.1002/smo2.70100

**Published:** 2026-08-28

**Authors:** Di Zhao, Zhizhong Wang, Ke Xu, Yuntai Liu, Di Wu, Ting Jiang, Haonan Gao, Chengmei Xiao, Debin Zheng, Kai Wang, Wenhao Zhai

**Affiliations:** ^1^ College of Pharmacy, Development and Utilization of Medicinal Resources in Liupanshan Area Ministry of Education Ningxia Medical University Yinchuan China; ^2^ Medicine Medical Innovation Research Division and First Medical Center of the Chinese PLA General Hospital Beijing China; ^3^ School of Pharmaceutical Sciences & Institute of Materia Medica, Shandong First Medical University & Shandong Academy of Medical Sciences Jinan China

**Keywords:** dual‐responsive, fluorescent probe, NAFLD, polarity, viscosity

## Abstract

Viscosity and polarity are critical physiological parameters implicated in the onset and progression of nonalcoholic fatty liver disease (NAFLD). Real‐time monitoring of their dynamic variations is of significant importance for the early diagnosis of NAFLD. In this study, BODIPY was utilized as the fluorophore core to successfully develop a dual‐responsive fluorescent probe, ^***Mp***^ ***BDP‐CF***_***3***_, through the incorporation of a trifluoromethyl (CF_3_) group at the meso position and methoxyphenyl groups at the 3,5‐positions. The probe demonstrated a high fluorescence quantum yield (approximately 47.86% in 1,4‐dioxane) and excellent photostability. It exhibited remarkable fluorescence sensitivity to variations in both viscosity and polarity within solution and cellular environments, achieving a detection limit as low as 0.41 cP for viscosity. Furthermore, ^***Mp***^ ***BDP‐CF***_***3***_ demonstrated effective targeting of lipid droplets during NAFLD progression. Subsequent in vivo investigations revealed that ^***Mp***^ ***BDP‐CF***_***3***_ facilitated high‐contrast fluorescence imaging and precise discrimination of NAFLD in BALB/c nude mouse models, underscoring its substantial potential for early NAFLD diagnosis.

## Graphical abstract

We developed a BODIPY‐based fluorescent probe with dual responsiveness to viscosity and polarity, enabling sensitive and precise imaging of non‐alcoholic fatty liver disease.

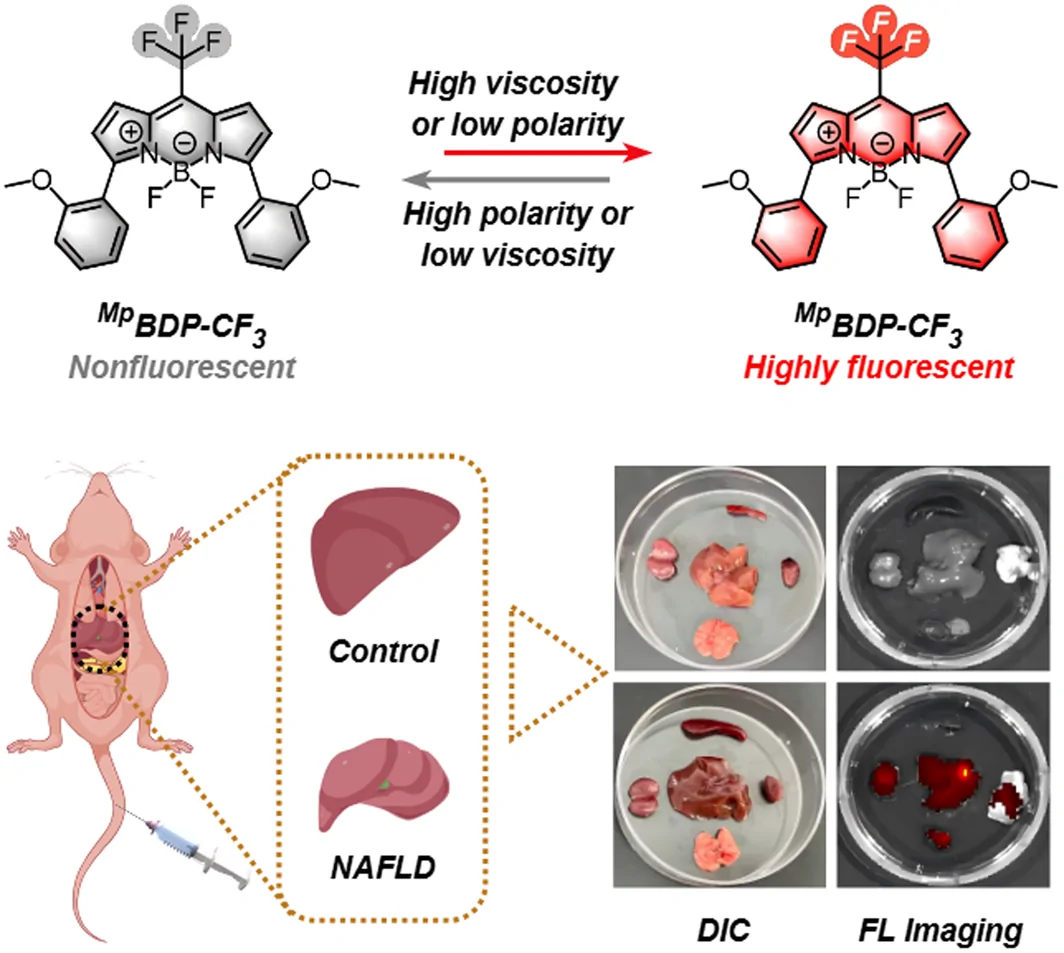

## 1 INTRODUCTION

Nonalcoholic fatty liver disease (NAFLD) has become the most common chronic liver condition globally. Its progression may result in liver fibrosis, cirrhosis, and in severe instances, hepatocellular carcinoma.[^1^, ^2^, ^3^, ^4^] Despite the rising prevalence and significant health impact of NAFLD, its pathogenesis is not yet fully elucidated, and no targeted therapeutic strategies exist to halt or reverse disease progression.[^5^, ^6^] Consequently, early diagnosis and risk stratification are critically important for preventing the advancement of NAFLD. Currently, liver biopsy is considered the clinical gold standard for diagnosing NAFLD. However, its broad application is constrained by several inherent limitations, such as invasiveness, sampling bias, procedural complexity, time requirements, and low patient compliance.[^7^, ^8^, ^9^, ^10^] Therefore, there is a strong need to develop noninvasive, sensitive, and real‐time diagnostic methods for NAFLD.

Fluorescence imaging has garnered significant interest in disease diagnosis due to its high sensitivity, superior spatial resolution, and noninvasive characteristics.[^11^, ^12^, ^13^] In this context, fluorescent probes that respond to disease‐associated biomarkers or alterations in the microenvironment have shown considerable potential for the diagnosis of NAFLD. NAFLD is a prototypical metabolic disorder characterized by impaired lipid metabolism within hepatocytes, leading to the progressive accumulation of lipid droplets (LDs) and concomitant modifications in the intracellular microenvironment, including increased viscosity and altered polarity.[^14^, ^15^, ^16^, ^17^] Consequently, the visualization of LDs alongside their associated microenvironmental parameters, such as viscosity and polarity, is critically important for early diagnosis, monitoring disease progression, and elucidating the underlying mechanisms of NAFLD. In recent years, considerable efforts have been directed toward the development of fluorescent probes capable of detecting microenvironmental changes associated with NAFLD. For instance, Chen et al. reported a sulfone‐substituted D‐π‐A fluorophore based on dicyanomethylene‐4H‐chromene, which effectively visualizes viscosity changes during NAFLD‐related ferroptosis and demonstrates favorable sensing performance.^[18]^ Furthermore, Wu et al., as well as Li et al., developed the LDs‐targeting fluorescent probe ANI^[19]^ and the polarity‐ultrasensitive fluorescent probe Lipi‐NIFD,^[20]^ respectively, which enable LDs imaging and the quantitative assessment of polarity changes in hepatic LDs. These studies have contributed valuable molecular imaging tools for investigating liver diseases. To provide a comprehensive overview of recent advancements in this area, we systematically reviewed the reported fluorescent probes for the diagnosis of NAFLD (Supporting Information S1: Table S1). Collectively, these findings indicate that the simultaneous monitoring of viscosity and polarity holds promise for offering a more thorough and precise understanding of the early pathological processes of NAFLD, thereby facilitating its early diagnosis and mechanistic exploration.

**FIGURE 1:**
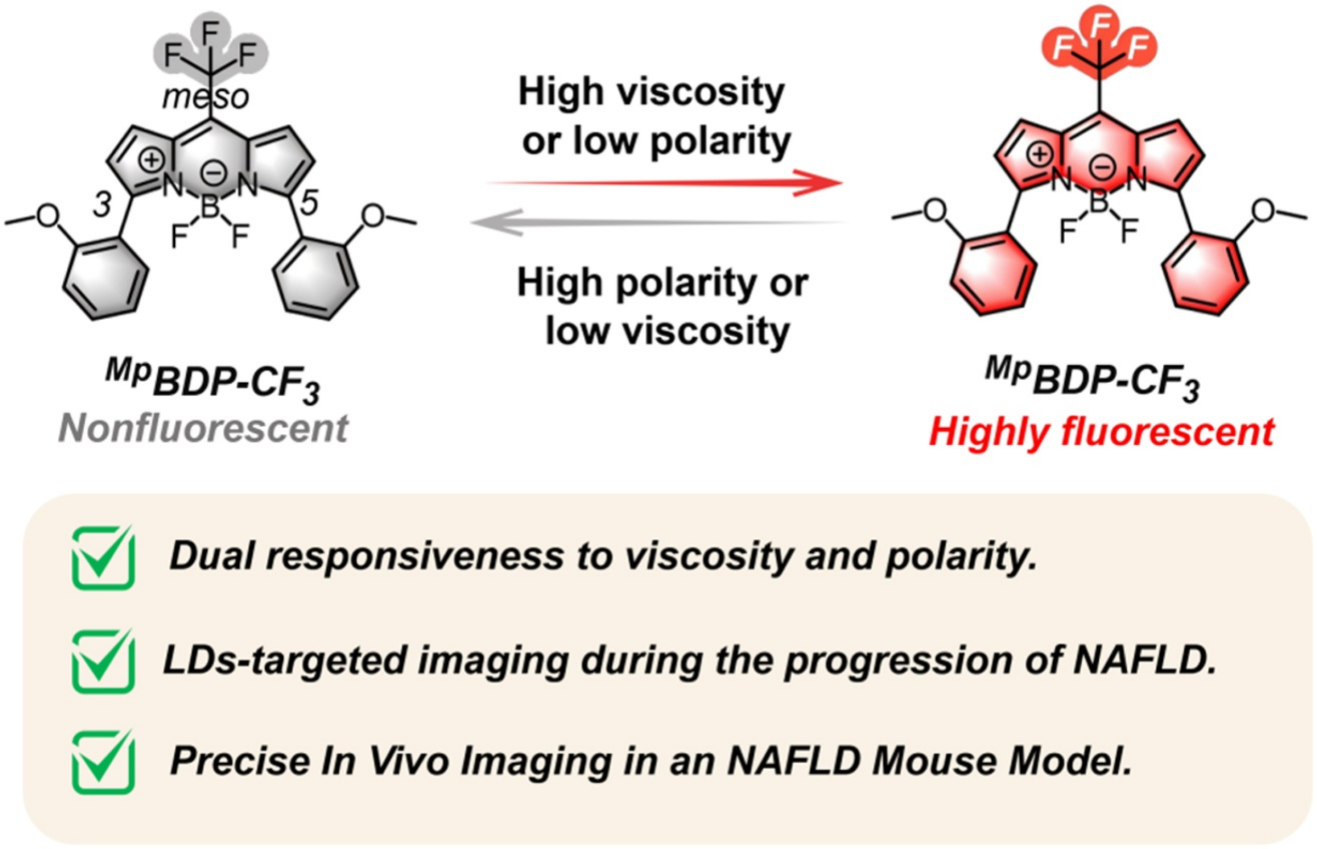
Mechanistic illustration of ^***Mp***^ ***BDP‐CF***_***3***_ for viscosity and polarity sensing.

Motivated by the aforementioned considerations, we designed and synthesized a viscosity‐ and polarity‐dual‐responsive fluorescent probe, ^***Mp***^ ***BDP‐CF***_***3***_, utilizing BODIPY as the fluorophore scaffold, with a CF_3_ group introduced at the meso position and methoxyphenyl substituents at the 3,5‐positions (Figure 1). ^***Mp***^ ***BDP‐CF***_***3***_ demonstrated a high fluorescence quantum yield (47.86% in 1,4‐dioxane) alongside excellent photostability, rendering it suitable for long‐term biological imaging applications. Leveraging the synergistic detection of viscosity and polarity, we hypothesized that ^***Mp***^ ***BDP‐CF***_***3***_ could facilitate precise monitoring of the microenvironment associated with NAFLD, thereby offering a promising approach for early diagnosis and timely therapeutic intervention. Notably, ^***Mp***^ ***BDP‐CF***_***3***_ exhibited high sensitivity to variations in both viscosity and polarity in solution and within living cells, confirming its excellent imaging capabilities. Importantly, the probe demonstrated remarkable targeting specificity toward LDs, which we attribute to the inherent lipophilicity of ^***Mp***^ ***BDP‐CF***_***3***_'s molecular structure.^[21]^ Moreover, ^***Mp***^ ***BDP‐CF***_***3***_ enabled real‐time fluorescence imaging of the liver in a murine NAFLD model, underscoring its potential as a molecular imaging tool for noninvasive early diagnosis and dynamic monitoring of disease progression.

## 2 RESULTS AND DISCUSSION

### 2.1 Molecular synthesis and theoretical calculations

**FIGURE 2:**
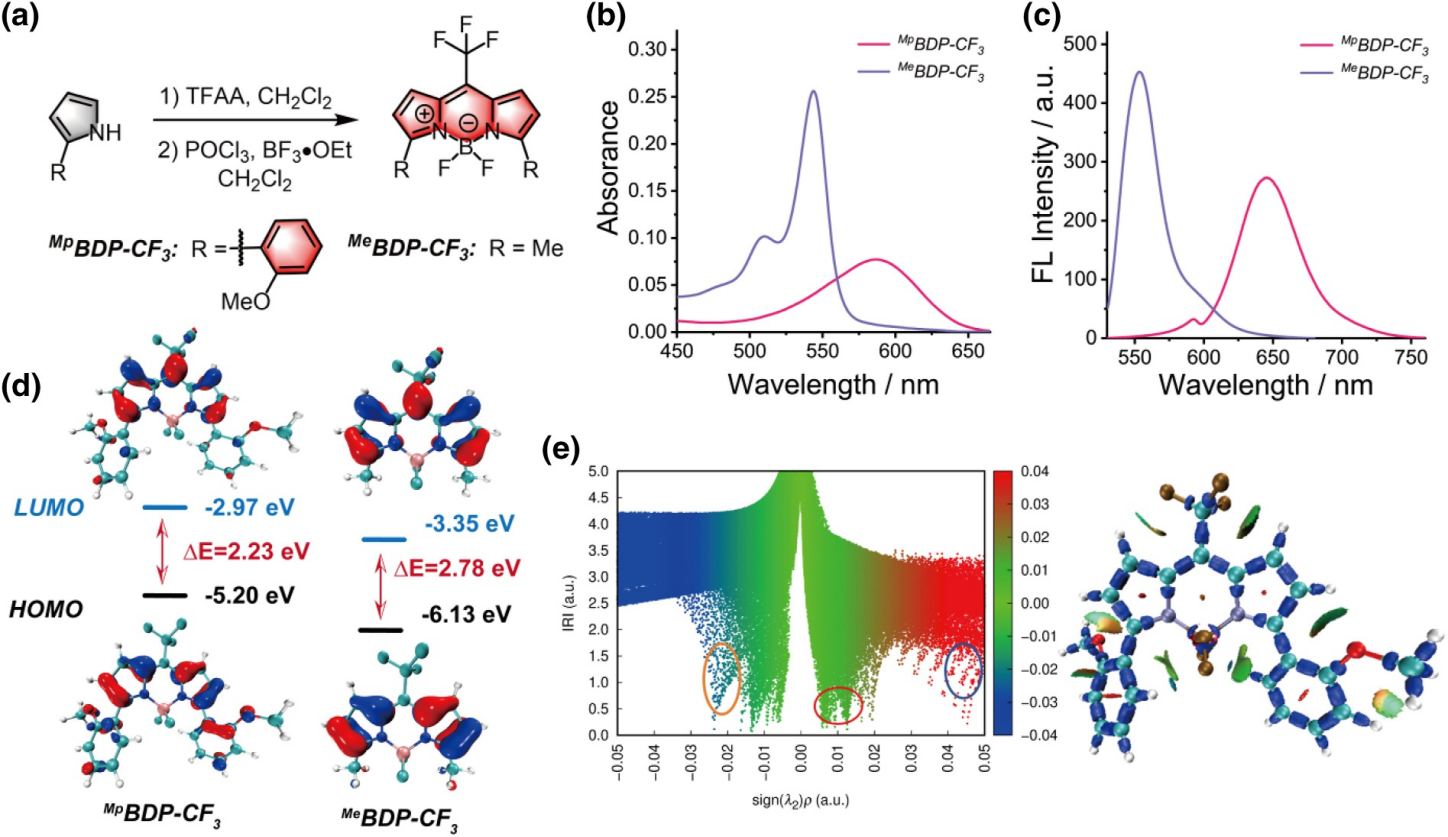
(a) Synthetic routes and chemical structures of ^Me^BDP‐CF_3_ and ^***Mp***^ ***BDP‐CF***_***3***_. (b) UV‐Vis absorption spectra and (c) fluorescence emission spectra recorded for 2.0 μM solution of ^Me^BDP‐CF_3_ and ^***Mp***^ ***BDP‐CF***_***3***_ at 37°C. (d) Frontier molecular orbitals and energies of ^Me^BDP‐CF_3_ and ^***Mp***^ ***BDP‐CF***_***3***_ from DFT calculations. (e) Scatter plot and visualization of interaction region index in ^***Mp***^ ***BDP‐CF***_***3***_. DFT, density functional theory.

^Me^BDP‐CF_3_ and ^***Mp***^ ***BDP‐CF***_***3***_ were synthesized following the synthetic route depicted in Figure 2a, with detailed procedures provided in the Supporting Information. All key intermediates and target compounds were thoroughly characterized using ^1^H NMR (Supporting Information S1: Figures S10, S12 and S14), ^13^C NMR (Supporting Information S1: Figures S11, S13 and S15), and high‐resolution mass spectrometry (Supporting Information S1: Figure S16).

The photophysical properties of the two probes were subsequently examined in dimethyl sulfoxide (DMSO) solution. As illustrated in Figure 2b,c, ^Me^BDP‐CF_3_ and ^***Mp***^ ***BDP‐CF***_***3***_ exhibited maximum absorption wavelengths at 544 and 587 nm, respectively, with corresponding emission maxima at 553 and 645 nm. These experimental findings were consistent with the theoretically calculated values presented in Supporting Information S1: Figure S1. Compared to ^Me^BDP‐CF_3_, ^***Mp***^ ***BDP‐CF***_***3***_ demonstrated a significant red shift of approximately 43 nm in the absorption spectrum, as well as a substantial Stokes shift of approximately 60 nm.

Large Stokes shift is highly advantageous as it effectively minimizes the spectral overlap between excitation and emission, thereby reducing background interference and self‐absorption effects. This enhancement improves the signal‐to‐background ratio and detection sensitivity, ultimately facilitating fluorescence imaging in complex biological environments.[^22^, ^23^, ^24^] To further investigate the origin of the spectral red shift, density functional theory calculations were conducted. As illustrated in Figure 2d, increasing the electron‐donating capacity of the substituents at the 3,5‐positions from ^Me^BDP‐CF_3_ to ^***Mp***^ ***BDP‐CF***_***3***_ led to a notable elevation in both the highest occupied molecular orbital (HOMO) and lowest unoccupied molecular orbital (LUMO) energy levels, accompanied by a gradual decrease in the HOMO‐LUMO energy gap. The reduced energy gap induced a red shift in both the absorption and emission spectra, which aligns well with the experimental photophysical data.

Previous studies have demonstrated that CF_3_ groups function as typical molecular rotors, facilitating the development of viscosity‐sensitive fluorescent probes.[^25^, ^26^] Building upon this property, we conducted an interaction region indicator analysis of ^Me^BDP‐CF_3_ and ^***Mp***^ ***BDP‐CF***_***3***_ using the Multiwfn program to examine their intramolecular noncovalent interaction characteristics. As illustrated in Figure 2e and Supporting Information S1: Figure S2, the blue, green, and red regions within the scatter plots represent strong attractive interactions (such as hydrogen bonding or halogen bonding), van der Waals interactions, and steric repulsive interactions, respectively. Notably, the plots are predominantly characterized by prominent green spikes, indicating that van der Waals interactions constitute the primary noncovalent forces influencing the molecular conformations of both probes. These cooperative van der Waals interactions contribute to increased conformational rigidity of the molecular framework, thereby restricting the free rotation of the CF_3_ and suppressing intramolecular nonradiative relaxation processes. Consequently, the dissipation of excited‐state energy is diminished, resulting in enhanced fluorescence emission efficiency.

The restricted intramolecular motion characteristic confers high sensitivity to the probes with respect to microenvironmental changes. As environmental viscosity increases, intramolecular rotation is further impeded, effectively inhibiting nonradiative decay pathways and leading to a substantial enhancement in fluorescence intensity. Moreover, variations in environmental polarity can alter the molecular charge distribution and excited‐state energy levels, thereby further modulating the fluorescence behavior of the probes. Consequently, both ^Me^BDP‐CF_3_ and ^***Mp***^ ***BDP‐CF***_***3***_ are anticipated to function as dual‐responsive fluorescent probes capable of detecting changes in viscosity and polarity, rendering them promising candidates for dynamic imaging and monitoring of complex biological microenvironments.

### 2.2 Photophysical properties

Initially, the photophysical properties of ^Me^BDP‐CF_3_ and ^***Mp***^ ***BDP‐CF***_***3***_ were systematically examined across various solvent systems, with their UV‐Vis absorption and fluorescence emission spectra recorded. As illustrated in Supporting Information S1: Figure S3, ^***Mp***^ ***BDP‐CF***_***3***_ demonstrated markedly distinct absorption and emission behaviors depending on the solvent, reflecting its pronounced sensitivity to microenvironmental variations. Notably, ^***Mp***^ ***BDP‐CF***_***3***_ exhibited negligible fluorescence emission in phosphate buffered saline (PBS) aqueous solution (with a Φ_fl_ of approximately 0.06%), whereas significantly enhanced fluorescence signals were detected in highly viscous glycerol (with a Φ_fl_ of approximately 17.02%) and low‐polarity 1,4‐dioxane (with a Φ_fl_ of approximately 47.86%). Additional data on fluorescence quantum yield and molar extinction coefficients are presented in Supporting Information S1: Table S2. In contrast, ^Me^BDP‐CF_3_ did not display comparable fluorescence response characteristics. This observed difference can be attributed to the insufficiency of the CF_3_ group at the meso‐position alone to elicit a pronounced environmental response. The incorporation of methoxyphenyl groups at the 3,5‐positions not only enhances the molecule's electron‐donating capacity but also modulates intramolecular charge‐transfer processes and rotor dynamics, thereby substantially increasing the probe's sensitivity to microenvironmental variations. These findings indicate that the fluorescence emission of ^***Mp***^ ***BDP‐CF***_***3***_ is synergistically influenced by both environmental viscosity and polarity, underscoring its potential as a dual‐responsive fluorescent probe for imaging complex biological microenvironments.

**FIGURE 3:**
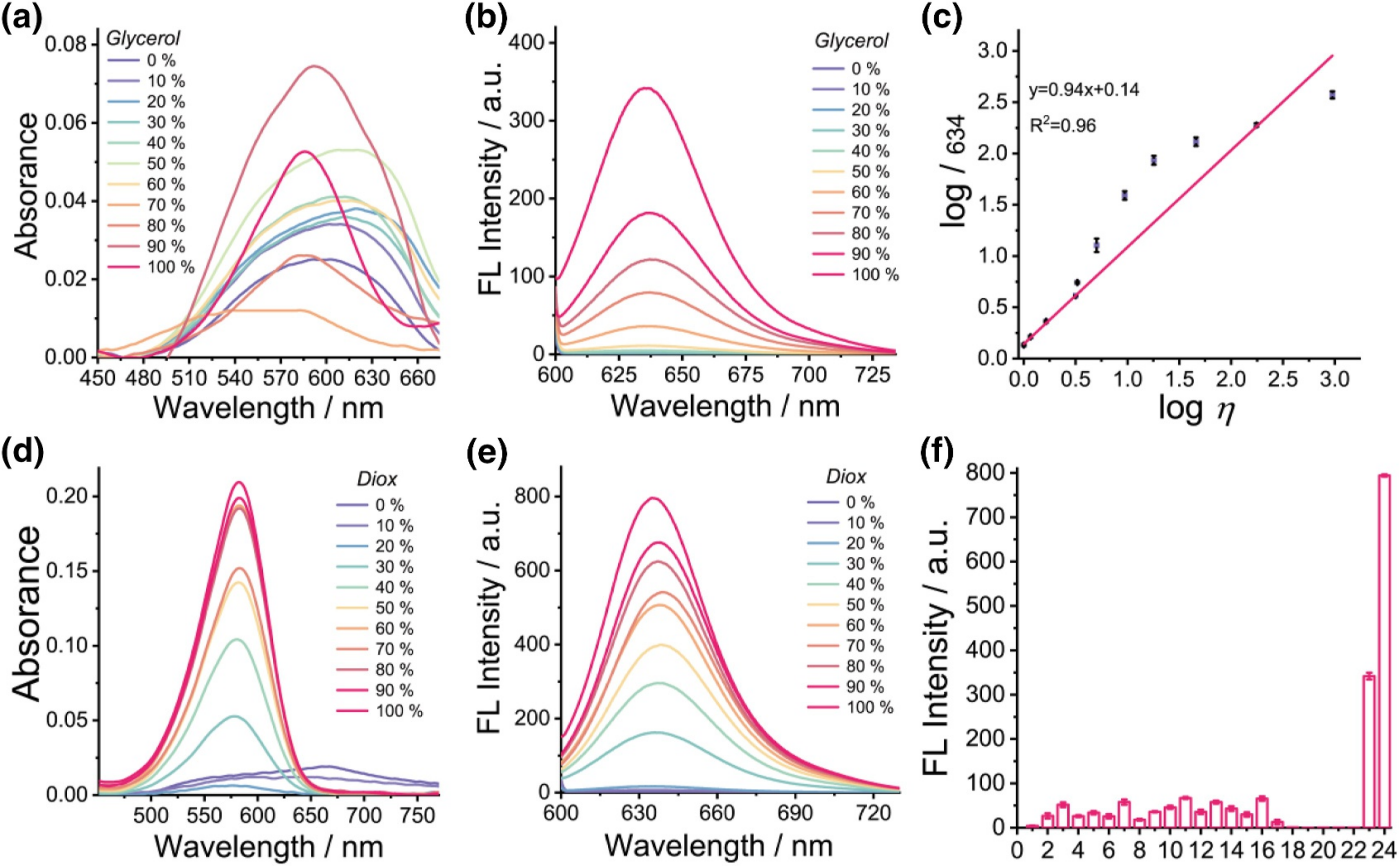
(a) UV‐Vis absorption spectra and (b) fluorescence emission spectra of ^***Mp***^ ***BDP‐CF***_***3***_ (2.0 μM) in glycerol/PBS mixtures of various fractions (0%–100%). (c) Linear fitted curve of log/634 versus log *η* for ^***Mp***^ ***BDP‐CF***_***3***_ (*n* = 3). (d) UV‐Vis absorption spectra and (e) fluorescence emission spectra of ^***Mp***^ ***BDP‐CF***_***3***_ (2.0 μM) in 1,4‐dioxane/PBS mixtures of various fractions (0%–100%). (f) Fluorescence response of ^***Mp***^ ***BDP‐CF***_***3***_ (2.0 μM) toward biological molecules (10 μM): 1: Blank, 2: Ala, 3: Cys, 4: Ile, 5: Lys, 6: Met, 7: Arg, 8: Ser, 9: Ag^+^, 10: Ca^2+^, 11: Cl^−^, 12: Cu^2+^, 13: Fe^3+^, 14: ${\text{HCO}}_{3}^{-}$, 15: ${\text{HSO}}_{4}^{-}$, 16: K^+^, 17: Mg^2+^, 18: cholesterol, 19: glucose, 20: H_2_O_2_, 21: NADH, 22: Cyt c, 23: glycerol, 24: 1,4‐dioxane (*n* = 3). NADH, nicotinamide adenine dinucleotide; PBS, phosphate buffered salin.

Consequently, we conducted a systematic investigation of the viscosity‐responsive behavior of ^***Mp***^ ***BDP‐CF***_***3***_ in glycerol/PBS mixtures with varying volume fractions, recording the corresponding UV–vis absorption and fluorescence emission spectra (Figure 3a,b). The results demonstrated that both probes exhibited minimal or nearly undetectable fluorescence emission in low‐viscosity aqueous solutions. As the glycerol content increased, resulting in a progressive rise in solution viscosity, the fluorescence emission bands centered at 544 and 587 nm were significantly enhanced, exhibiting a characteristic “turn‐on” fluorescence response. Furthermore, this fluorescence enhancement was readily observable to the naked eye (Supporting Information S1: Figure S5a), thereby corroborating that the emission behavior of both probes is closely linked to intramolecular rotor motion.

As illustrated in Figure 3c, the fluorescence intensity variations of both probes demonstrated strong linear correlations with solution viscosity, yielding excellent linear fitting results across a wide viscosity range of 1–945 cP (*R* ^2^ = 0.96). These linear responses suggest that ^***Mp***^ ***BDP‐CF***_***3***_ is capable of quantitatively detecting changes in microenvironmental viscosity. The limit of detection (LOD) was subsequently determined using the equation LOD = 3*δ*/S, where S represents the slope of the calibration curve and *δ* denotes the standard deviation of the intercept. The findings indicated that the LOD values for ^***Mp***^ ***BDP‐CF***_***3***_ was as low as 0.41 cP, underscoring their high sensitivity for viscosity sensing. Importantly, ^***Mp***^ ***BDP‐CF***_***3***_ exhibited a more pronounced response within the low‐viscosity range, emphasizing its enhanced ability to monitor subtle viscosity variations in biological systems in real time.

Subsequently, we conducted a systematic investigation of the polarity‐responsive behavior of ^***Mp***^ ***BDP‐CF***_***3***_ in mixtures of 1,4‐dioxane and PBS with varying volume fractions. The corresponding UV‐Vis absorption and fluorescence emission spectra were recorded (Figure 3d,e and Supporting Information S1: Figure S4a,b). The results demonstrated that both probes exhibited minimal or nearly undetectable fluorescence emission in highly polar aqueous environment. As the proportion of 1,4‐dioxane increased, resulting in a reduction of solvent polarity, the fluorescence intensity of ^***Mp***^ ***BDP‐CF***_***3***_ at 635 nm increased progressively, indicating a pronounced polarity‐dependent fluorescence response. Further analysis demonstrated a strong negative linear correlation between the maximum emission wavelength of ^***Mp***^ ***BDP‐CF***_***3***_ and the solvent polarity parameter (Δ*f*), with a linear correlation coefficient (*R* ^2^) of 0.87 (Supporting Information S1: Figure S4c). This finding indicates that a decrease in environmental polarity substantially influences the excited‐state energy distribution and the intramolecular charge‐transfer process, thereby effectively modulating the emission behavior and confirming the probe's excellent polarity‐sensing capability. Consequently, ^***Mp***^ ***BDP‐CF***_***3***_ exhibits a reliable linear response to polarity changes and can serve as a robust fluorescent indicator for monitoring polarity variations in complex microenvironments.

Notably, selectivity experiments further demonstrated that both probes exhibit excellent responsiveness to changes in viscosity and polarity, as well as high environmental sensitivity, while displaying minimal interference from other common biologically relevant species (Figure 3f). These dual‐responsive properties confer significant potential for the real‐time monitoring of microenvironmental viscosity and polarity dynamics within complex biological systems, thereby providing a powerful tool for imaging disease‐related microenvironments and visualizing biological processes.

### 2.3 Imaging of intracellular viscosity and polarity

Given the excellent viscosity‐ and polarity‐responsive properties of ^***Mp***^ ***BDP‐CF***_***3***_ in solution, its potential for intracellular microenvironment imaging was further investigated. Initially, the cytotoxicity of ^***Mp***^ ***BDP‐CF***_***3***_ was systematically evaluated in normal NIH 3T3 cells and hepatocellular carcinoma HepG2 cells using a standard CCK‐8 assay (Supporting Information S1: Figure S6). The results demonstrated that even at relatively high concentrations (60 μM), both NIH 3T3 and HepG2 cells maintained high viability following treatment with ^***Mp***^ ***BDP‐CF***_***3***_, with cell survival rates exceeding 90%. These findings suggest that ^***Mp***^ ***BDP‐CF***_***3***_ possesses good biocompatibility and low cytotoxicity, exhibiting no significant toxic effects on either normal or cancerous cells, thereby establishing a robust basis for subsequent bioimaging applications.

**FIGURE 4:**
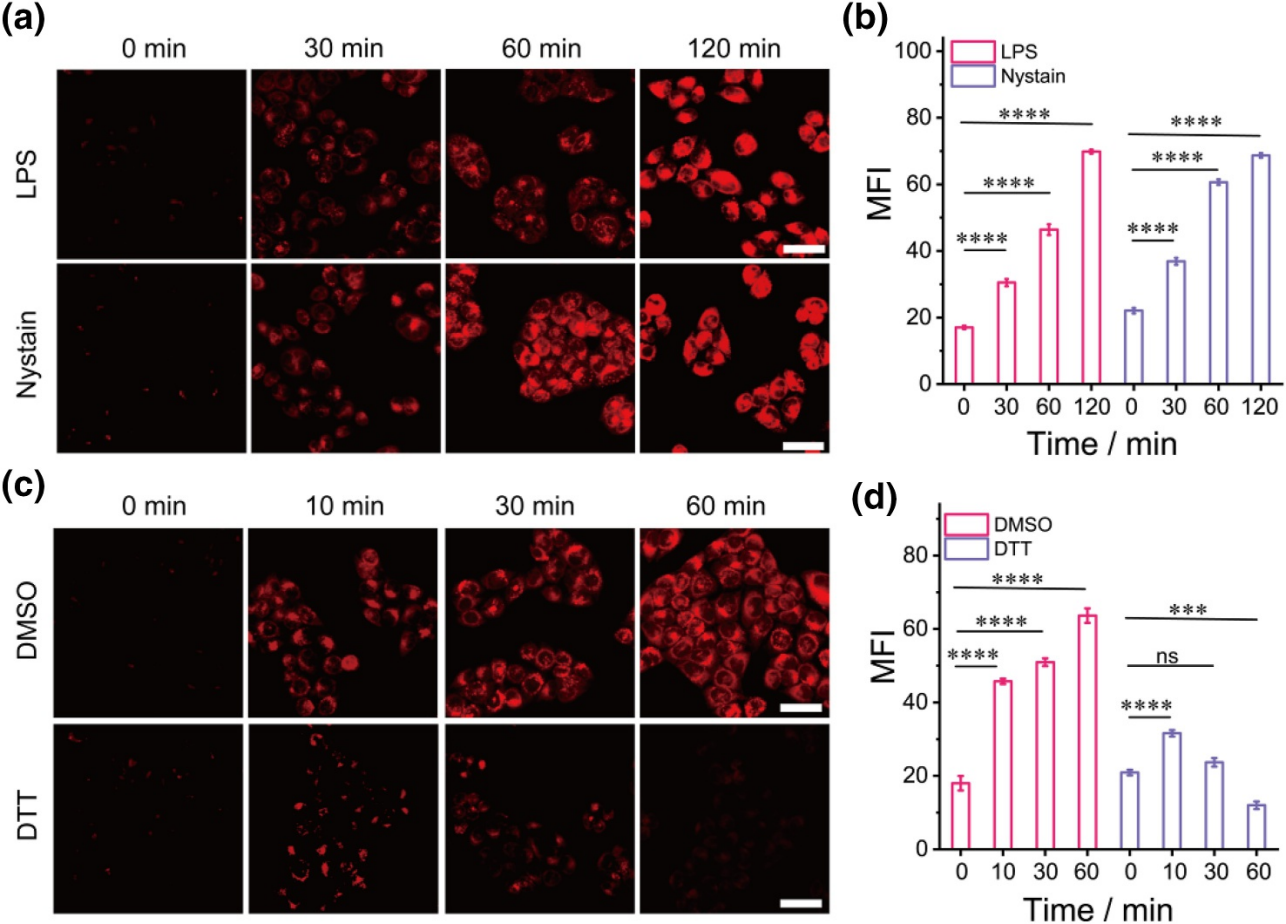
HepG2 cells were pretreated with (a) LPS (200 μg/mL) and Nystatin (20 μM), (c) DMSO (10 μL/mL) and DTT (5 mM) for different durations (0, 30, 60, and 120 min), followed by incubation with 2.0 μM ^***Mp***^ ***BDP‐CF***_***3***_ for 30 min prior to fluorescence imaging. (b, d) Present the quantitative analysis of the mean fluorescence intensity corresponding to the cellular images displayed in (a, c), respectively (*n* = 3). Scale bar: 50 μm. One‐way ANOVA, mean ± SD, ***p < 0.001, ****p < 0.0001, ns represents no significance. DMSO, dimethyl sulfoxide; DTT, dithiothreitol; LPS, lipopolysaccharide.

To assess the capability of ^***Mp***^ ***BDP‐CF***_***3***_ in detecting intracellular viscosity changes, lipopolysaccharide (LPS) and nystatin were utilized as model agents to modulate the intracellular microenvironmental viscosity. These compounds have been extensively documented to rapidly elevate intracellular viscosity by inducing cellular stress and altering membrane structures, and are therefore commonly employed to validate viscosity‐responsive fluorescent probes.[^27^, ^28^] As illustrated in Figure 4a, the untreated control group exhibited only very weak or nearly negligible red fluorescence signals, indicating that ^***Mp***^ ***BDP‐CF***_***3***_ remains in a low‐emissive state under normal cellular conditions. In contrast, cells treated with LPS (200 μg/mL) or nystatin (20 μM) demonstrated a significant enhancement of red fluorescence as early as 30 min post‐treatment. Furthermore, the fluorescence intensity increased in a time‐dependent manner with prolonged incubation. Quantitative analysis presented in Figure 4b demonstrated that following 2 h of treatment with LPS and nystatin, the intracellular fluorescence intensity increased by approximately 3.5‐fold and 3.2‐fold, respectively, relative to the control group. This finding indicates that ^***Mp***^ ***BDP‐CF***_***3***_ can sensitively detect elevations in intracellular viscosity. To further assess the probe's sensitivity, the concentrations of LPS and nystatin were reduced tenfold. As illustrated in Supporting Information S1: Figure S7a,b, even at lower concentrations of LPS (20 μg/mL) and nystatin (2 μM), ^***Mp***^ ***BDP‐CF***_***3***_ exhibited a significant fluorescence enhancement, with the signal progressively increasing over time, demonstrating a clear time‐dependent trend. These results suggest that ^***Mp***^ ***BDP‐CF***_***3***_ possesses high sensitivity to subtle intracellular viscosity changes and excellent dynamic monitoring capabilities, underscoring its potential as a high‐performance viscosity probe for bioimaging and investigation of disease‐related microenvironments.

Subsequently, we assessed the responsiveness of ^***Mp***^ ***BDP‐CF***_***3***_ to alterations in intracellular polarity at the cellular level. DMSO and dithiothreitol (DTT) were employed as representative agents for modulating polarity. DMSO rapidly reduces the polarity of the cellular microenvironment, whereas DTT increases intracellular polarity by cleaving disulfide bonds in proteins, thereby inducing conformational changes in protein structures.[^29^, ^30^] As illustrated in Figure 4c, treatment of HepG2 cells with DMSO (10 μL/mL) resulted in a marked difference in fluorescence between the control and treated groups, observable as early as 10 min post‐treatment. With prolonged incubation, the intracellular red fluorescence intensity progressively increased, demonstrating a clear time‐dependent pattern. Quantitative analysis (Figure 4d) revealed that after 60 min of treatment, the relative fluorescence intensity in the DMSO‐treated group was approximately 2.6 times greater than that of the control group. Notably, even when the DMSO concentration was reduced to 5 μL/mL, a comparable trend of fluorescence enhancement was observed (Supporting Information S1: Figure S8a,b). These findings are consistent with the “low‐polarity‐induced fluorescence enhancement” previously reported in solution studies, suggesting that ^***Mp***^ ***BDP‐CF***_***3***_ is highly sensitive to reductions in intracellular polarity.

In contrast, as illustrated in Figure 4c, cells treated with 5 mM DTT exhibited a fluorescence response characterized by an initial increase followed by a gradual decrease. With prolonged incubation, the intracellular fluorescence intensity progressively declined, decreasing by approximately 52% after 1 h of treatment compared to the control group. A similar trend was observed at lower DTT concentrations (Supporting Information S1: Figure S8a,b). These findings further demonstrate that ^***Mp***^ ***BDP‐CF***_***3***_ effectively responds to increases in intracellular polarity.

### 2.4 Cellular imaging of LDs

**FIGURE 5:**
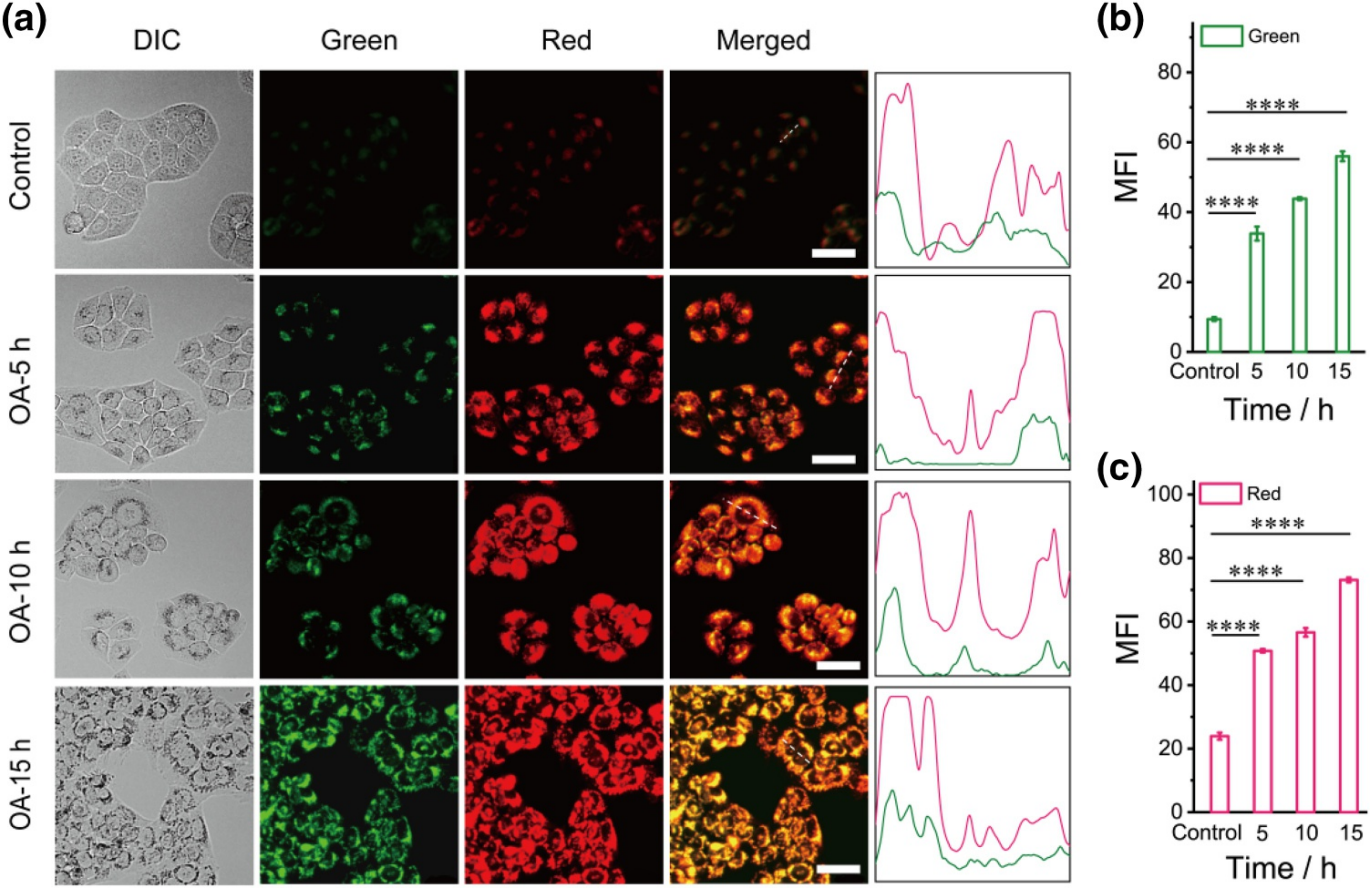
(a) HepG2 cells were pretreated with 500 μM oleic acid for 5, 10, and 15 h, respectively, followed by incubation with a staining solution containing 2.0 μM BODIPY 493/503 and 2.0 μM ^***Mp***^ ***BDP‐CF***_***3***_ for 30 min prior to fluorescence imaging. Scale bar: 50 μm. Quantitative analysis of the average fluorescence intensities in the (b) green and (c) red channels is presented in (a) (*n* = 3). One‐way ANOVA, mean ± SD, ****p < 0.0001, ns represents no significance.

LDs are essential organelles involved in intracellular lipid metabolism and fat storage. Alterations in their quantity and physicochemical characteristics are closely linked to the onset and progression of NAFLD.[^31^, ^32^] Consequently, precise monitoring of the dynamic changes in LDs is critically important for enhancing the early diagnosis and disease staging of NAFLD. Building upon the excellent viscosity and polarity responsive properties of ^***Mp***^ ***BDP‐CF***_***3***_, we further investigated its LDs targeting capability and microenvironmental responsiveness within a NAFLD cellular model. In this study, HepG2 cells were treated with 500 μM oleic acid (OA) to induce lipid accumulation. OA facilitates intracellular LDs formation, thereby simulating lipid metabolic disorders associated with NAFLD. As illustrated in Figure 5a, compared to the control group, HepG2 cells treated with OA demonstrated a significant increase in the number of LDs, with accumulation becoming more pronounced over extended incubation periods, consistent with previous findings.^[33]^ To further confirm the specific localization of ^***Mp***^ ***BDP‐CF***_***3***_ to LDs, colocalization experiments were conducted by co‐incubating model cells with the commercial LDs probe BODIPY 493/503 and ^***Mp***^ ***BDP‐CF***_***3***_ for 30 min. The red fluorescence emitted by ^***Mp***^ ***BDP‐CF***_***3***_ demonstrated substantial spatial overlap with the green fluorescence of BODIPY 493/503, indicating a strong LDs‐targeting capability of ^***Mp***^ ***BDP‐CF***_***3***_. Notably, the red fluorescence signal of ^***Mp***^ ***BDP‐CF***_***3***_ increased progressively with longer OA incubation time, revealing a clear time‐dependent and spatially resolved distribution pattern.

Quantitative analysis of mean cellular fluorescence intensity (Figure 5c) demonstrated that, relative to the control group, red fluorescence intensity increased by approximately 2.0‐fold at 5 h, 2.3‐fold at 10 h, and further reached 2.8‐fold at 15 h. Concurrently, the green fluorescence intensity of BODIPY 493/503 also exhibited a progressive increase with prolonged OA treatment, following a similar time‐dependent pattern. As illustrated in Supporting Information S1: Figure S9, comparable results were observed in normal hepatocytes under the same experimental conditions. OA‐induced LDs accumulation results in elevated microenvironmental viscosity and reduced local polarity within LDs.^[34]^ The high‐viscosity environment effectively restricts the intramolecular rotational motion of ^***Mp***^ ***BDP‐CF***_***3***_, thereby suppressing nonradiative decay pathways, while the low‐polarity environment further diminishes excited‐state energy dissipation. These factors synergistically enhance fluorescence emission. Consequently, the observed time‐dependent fluorescence enhancement of ^***Mp***^ ***BDP‐CF***_***3***_ reflects its dual sensitivity to the high‐viscosity and low‐polarity microenvironment of LDs, underscoring its potential utility for the in vivo diagnosis of NAFLD.

### 2.5 In vivo imaging of NAFLD mouse models

**FIGURE 6:**
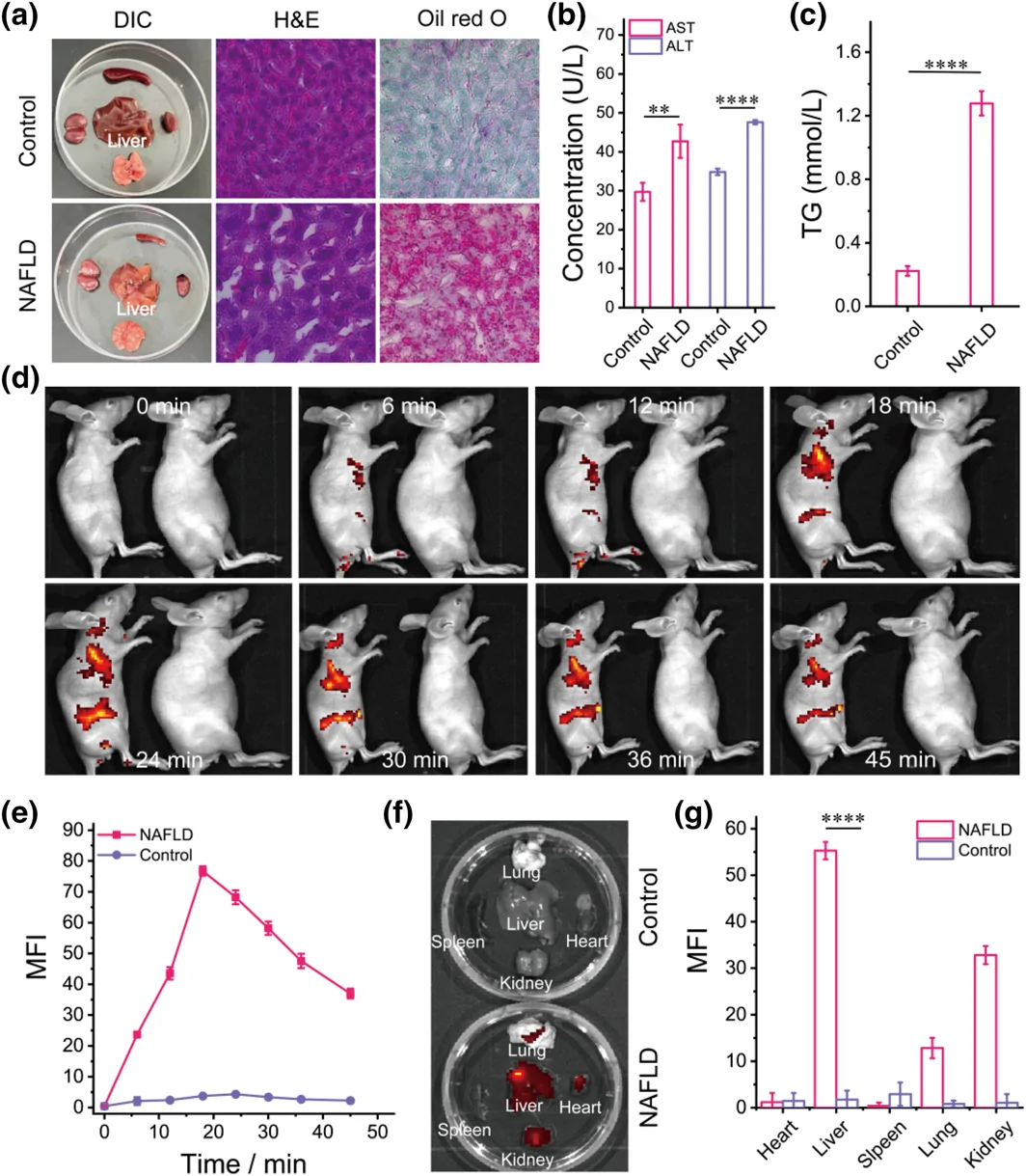
(a) Photographs of livers and liver tissues by H&E and Oil Red O staining in normal and NAFLD mice. (b) Serum AST/ALT levels and (c) hepatic TG contents in normal mice and NAFLD mice. (d) In vivo fluorescence imaging of NAFLD and normal mice was conducted at various time points following tail vein administration of ^***Mp***^ ***BDP‐CF***_***3***_ (200 μL, 5 mg/kg). (e) Quantitative analysis of the mean fluorescence intensity within the hepatic region of the mice depicted in (a) (*n* = 3). (f) Following in vivo imaging, the mice were euthanized, and their major organs were collected for ex vivo fluorescence imaging. (g) Quantitative analysis of the mean fluorescence intensity in ex vivo organs obtained from mice (*n* = 3). One‐way ANOVA, mean ± SD, **p < 0.01, ****p < 0.0001, ns represents no significance. ALT, alanine aminotransferase; AST, aspartate aminotransferase; NAFLD, nonalcoholic fatty liver disease; TG, triglyceride.

The outstanding viscosity and polarity dual‐responsive properties, along with the LDs‐targeting capability of ^***Mp***^ ***BDP‐CF***_***3***_ at the cellular level, motivated us to investigate its potential application for in vivo diagnosis of NAFLD. To this end, a BALB/c nude mouse model of NAFLD was established through a combination of a high‐fat diet and intraperitoneal administration of dexamethasone. In the experimental group, mice were fed a high‐fat diet and received intraperitoneal injections of dexamethasone (50 mg/kg) once every 2 days for a total of 14 d, whereas the control group mice were maintained on a standard diet. As illustrated in Figure 6a, one mouse from each of the model and control groups was randomly selected for dissection, and the major organs were subjected to morphological examination. Compared to normal mice, the NAFLD model mice displayed notably pale and yellowish livers, significantly enlarged liver size, rounded edges, and a conspicuously greasy surface. H&E staining revealed abnormal hepatic tissue architecture in NAFLD mice, characterized by disrupted hepatic cords and hepatocyte swelling. Oil Red O staining demonstrated significant lipid accumulation in the liver tissue of NAFLD mice (Figure 6a). Furthermore, serum levels of aspartate aminotransferase and alanine aminotransferase, as well as hepatic triglyceride content (Figure 6b,c), were significantly elevated in NAFLD mice compared with control mice. These characteristics are typical indicators of hepatic steatosis and align closely with previously reported pathological features of NAFLD,^[35]^ thereby confirming the successful establishment of the NAFLD mouse model.

Subsequently, both the model and control mice were intravenously administered ^***Mp***^ ***BDP‐CF***_***3***_ (200 μL, 5 mg/kg) via the tail vein, followed by in vivo fluorescence imaging. As illustrated in Figure 6a, the control mice exhibited negligible red fluorescence signals in the abdominal region. In contrast, the NAFLD model mice demonstrated a progressive increase in red fluorescence within the abdominal area following probe administration, reaching peak intensity approximately 20–30 min post‐injection, which was then followed by a gradual decline over time (Figure 6b). Ex vivo fluorescence imaging of dissected organs corroborated the in vivo imaging findings (Figure 6c). Compared to the control group, the NAFLD model group exhibited a markedly enhanced red fluorescence signal in the liver, with no significant fluorescence accumulation detected in other major organs. Quantitative analysis (Figure 6d) revealed that the relative fluorescence intensity in the livers of NAFLD mice was significantly greater than that observed in the control group, thereby further validating the high imaging sensitivity and liver‐specific recognition capability of ^***Mp***^ ***BDP‐CF***_***3***_ for NAFLD.

## 3 CONCLUSIONS

In summary, we have successfully developed a BODIPY‐based fluorescent probe, ^***Mp***^ ***BDP‐CF***_***3***_, exhibiting dual responsiveness to both viscosity and polarity. Through rational structural modifications, we effectively regulated intramolecular rotor motion and the electronic energy level structure, thereby enabling the simultaneous detection of changes in viscosity and polarity. ^***Mp***^ ***BDP‐CF***_***3***_ exhibits exceptional viscosity and polarity responsive properties in solution systems and facilitates the sensitive detection of subtle variations within intracellular microenvironments. Furthermore, it demonstrates a strong targeting capability toward LDs formed during the progression of NAFLD. Notably, ^***Mp***^ ***BDP‐CF***_***3***_ selectively accumulates in the liver region of BALB/c nude mice and generates significantly enhanced fluorescence signals in NAFLD models, underscoring its considerable potential for early diagnosis and fluorescence imaging of NAFLD.

## CONFLICT OF INTEREST STATEMENT

The authors declare no conflicts of interest.

## ETHICS STATEMENT

All animal experiments were performed following the protocols evaluated and approved by the Experimental Animal Ethics Committee of Ningxia Medical University (Permit No. IACUC‐2025331). Female BALB/c nude mice aged 6–8 weeks were purchased from Beijing Vital River Laboratory Animal Technology Co., Ltd. (Beijing, China).

## Supporting information

Supporting Information S1

## Data Availability

The data that support the findings of this study are available on request from the corresponding author. The data are not publicly available due to privacy or ethical restrictions.
