# Supplementary material for: A dual‐responsive fluorescent probe for imaging viscosity and polarity in nonalcoholic fatty liver disease
Source: Smart Mol. 2026 Aug 28:e70100. Online ahead of print. doi: 10.1002/smo2.70100 (PMC13525181; doi:10.1002/smo2.70100)
Supplement: Supplementary file 1 [file SMO2-9999-0-s001.docx]

# Supporting Information

**A dual-responsive fluorescent probe for imaging viscosity and polarity in nonalcoholic fatty liver disease**

*Di Zhao^1^, Zhizhong Wang^1,^*, Ke Xu^1^, Yuntai Liu^1^, Di Wu^3^, Ting Jiang^1^, Haonan Gao^1^, Chengmei Xiao^2,^*, Debin Zheng^3,^*, Kai Wang^2,^*, and Wenhao Zhai^1,^**

^1^College of Pharmacy, Development and Utilization of Medicinal Resources in Liupanshan Area, Ministry of Education, Ningxia Medical University, Yinchuan 750004, P. R. China.

^2^School of Pharmaceutical Sciences & Institute of Materia Medica, Shandong First Medical University & Shandong Academy of Medical Sciences, Jinan 250117, P. R. China.

^3^Medicine Medical Innovation Research Division and First Medical Center of the Chinese PLA General Hospital, No. 28 Fu Xing Road, Beijing 100853, P. R. China.

Corresponding author: Zhizhong Wang (wangzzsc@163.com), Chengmei Xiao (Xiaochengmei0301@163.com), Debin Zheng (zhengdebin301@163.com), Kai Wang (wangkaihello@foxmail.com), Wenhao Zhai (20220104@nxmu.edu.cn).

**Content**

[1. Materials and instruments	3](#_Toc14584)

[2. Chemical synthesis	4](#_Toc31059)

[3. Experimental Methods	5](#_Toc11764)

[3.1. Optical Characterization of](#_Toc15081) ***^[Mp](#_Toc15081)^[BDP-CF](#_Toc15081)_[3](#_Toc15081)_***	[5](#_Toc15081)

[3.2. Fluorescence quantum yield (](#_Toc12206)*[Φ](#_Toc12206)_[fl](#_Toc12206)_*[) determination	6](#_Toc12206)

[3.3. Theoretical calculation	6](#_Toc10051)

[3.4. Cell Culture	6](#_Toc2271)

[3.5. Cell viability assay.	7](#_Toc30335)

[3.6. Cellular viscosity imaging.	7](#_Toc26678)

[3.7. Cell polarity imaging.	7](#_Toc19841)

[3.8. NAFLD Imaging.	7](#_Toc28602)

[4. Establishment of the NAFLD Nude Mouse Model.	8](#_Toc20079)

[5. Supplementary Figures	9](#_Toc28600)

[6. NMR and HRMS spectra	15](#_Toc14934)

[7. References	18](#_Toc22560)

1. **Materials and instruments**

Unless otherwise noted, reagents were used as received from commercial sources. Dulbecco’s Modified Essential Medium (DMEM), RPMI-1640 medium, fetal bovine serum (FBS), and 10 × phosphate buffer saline (PBS; pH 7.4) were purchased from Gibco (Life Technologies, AG, Switzerland). Lipopolysaccharide (LPS), Nystatin, Oleic acid, Dimethyl sulfoxide, Dithiothreitol (DTT), and Fluorescent dye BODIPY 493/503 were purchased from Sigma-Aldrich. The AST, ALT, and TG assay kits were purchased from Nanjing Jiancheng Bioengineering Institute (Nanjing, China). Water was deionized with a Milli-Q SP reagent water system (Millipore) to a specific resistivity of 18.2 MΩ cm.^Me^BDP-CF_3_ was synthesized according to literature procedures^[1]^.

^1^H and ^13^C NMR spectra were recorded at 25ºC on a Bruker AV400 NMR spectrometer, operating at 400 and 100 MHz, respectively, where chemical shifts (*δ* in ppm) were determined using partially or non-deuterated solvent residues as internal references. CDCl_3_ was used as the solvent. High-resolution mass spectra (HRMS) were obtained on Varian 7.0 T FTMS. UV-visible absorption spectra were recorded on a UV2600 double-beam UV-Vis spectrophotometer (Shimadzu). Fluorescence spectra were recorded on an FL7100 (Hitachi) spectrofluorometer. CCK-8 assay was monitored by the microplate reader (SpectraMax i3x, MD). Inverted fluorescence microscope (IFM) images were acquired using Thermo Fisher Scientific. In Vivo Fluorescence imaging of tumor-bearing mice was conducted on NightOWL II LB983 In Vivo Imaging System (Berthold Technologies).

2. **Chemical synthesis**

***Compound 1***: To a dry toluene solution of PdCl₂(CH₃CN)₂ (5.18 mg, 0.02 mmol), MePhos (14.58 mg, 0.04 mmol) at 0ºC, pyrrole (0.14 mL, 2.0 mmol) was added under argon, and the mixture was stirred at this temperature for 1 h, followed by the addition of 2-chloroanisole (0.14 g, 1.0 mmol). The mixture was then heated to 110ºC and refluxed for 4 h. After the reaction mixture was cooled to room temperature, saturated brine (2.0 mL) and dichloromethane (10.0 mL) were added sequentially. The resulting solution was filtered through a celite pad and extracted with dichloromethane (20.0 mL×3). The combined organic layers were dried over anhydrous sodium sulfate and concentrated. The residues were further purified by silica gel column chromatography using PE/DCM (3/1, v/v) as eluent, affording the compound 1 as a light blue solid (0.14 g, 80.9%). ^1^H NMR (400 MHz, CDCl_3_, Figure S10) *δ* = 9.85 (s, 1 H), 7.70 (d, *J* = 7.60 Hz, 1 H), 7.21 - 7.16 (m, 1 H), 7.04 - 6.98 (m, 2 H), 6.89 (s, 1 H), 6.66 (s, 1 H), 6.32 (q, *J* = 2.8 Hz, 1 H), 3.97 (s, 3 H). ^13^C NMR (100 MHz, CDCl_3_, Figure S11) *δ* 154.72, 129.88, 126.71, 126.64, 121.47, 121.11, 117.83, 111.65, 108.87, 106.12, 55.70.

***Compound*** ***2***: To a dry dichloromethane (5.0 mL) solution of compound 1 (0.50 g, 2.87 mmol) at 0ºC, trifluoroacetic anhydride (0.44 mL, 3.1 mmol) was added dropwise under argon, and the mixture was stirred at this temperature for 30 min. The mixture was then warmed to room temperature and stirred for 3 h. After the reaction was completed, the reaction was quenched with saturated aqueous sodium bicarbonate solution and extracted with dichloromethane (20 mL×3). The combined organic layers were dried over anhydrous sodium sulfate and concentrated to remove the organic solvent. The residues were further purified by silica gel column chromatography using PE/DCM (2/1, v/v) as eluent, affording Compound 2 as a white solid (0.36 g, 46.5%). ^1^H NMR (400 MHz, CDCl_3_, Figure S12) *δ* = 10.78 (s, 1 H), 7.73 (d, *J* = 8.00 Hz, 1 H), 7.36 (t, *J* = 7.2 Hz, 1 H), 7.24 (s, 1 H), 7.09 - 7.04 (m, 2 H), 6.76 (q, *J* = 2.8 Hz, 1 H), 4.04 (s, 3 H). ^13^C NMR (100 MHz, CDCl_3_, Figure S13) *δ* 156.28, 140.48, 130.42, 128.39, 125.33, 121.92, 121.56, 118.71, 117.77, 115.84, 111.82, 110.34, 55.96.

***^Mp^BDP-CF_3_***: To a dry dichloromethane (5.0 mL) solution of compound 1 (0.38 g, 2.18 mmol) and compound 2 (0.4 g, 1.48 mmol) at 0ºC, phosphorus oxychloride (0.21 mL, 2.2 mmol) was added under argon. The mixture was warmed to room temperature and stirred for 4 h, followed by the sequential addition of triethylamine (1.03 mL, 7.4 mmol) and boron trifluoride diethyl etherate (0.94 mL, 7.4 mmol), and the mixture was stirred for another 2 h. After removing the solvent, the residues were further purified by silica gel column chromatography using PE/ethyl acetate (20/1, v/v) as eluent, affording the ***^Mp^BDP-CF_3_*** of red solid (0.12 g, 17.1%). ^1^H NMR (400 MHz, CDCl_3_, Figure S14) *δ* = 7.73 (d, *J* = 7.20 Hz, 2 H), 7.38 - 7.34 (m, 4 H), 7.00 (t, *J* = 7.6 Hz, 2 H), 6.92 (d, *J* = 8.40 Hz, 2 H), 6.71 (d, *J* = 4.8 Hz, 2 H), 3.78 (s, 6 H). ^13^C NMR (100 MHz, CDCl_3_, Figure S15) *δ* 158.66, 157.68, 132.50, 131.76, 131.71, 131.66, 131.39, 128.98, 124.34, 121.20, 120.33, 110.89, 55.75. HRMS (ESI, Figure S16) calc. for C24H_20_BF5N2O_2_ [M+Na]^+^ 495.1273, found 495.1274.

## 3. Experimental Methods

3.1. **Optical Characterization of *^Mp^BDP-CF_3_***

A stock solution of ***^Mp^BDP-CF_3_*** (2 mM) and ^Me^BDP-CF_3_ (2 mM) were prepared in DMSO, and the working solution was diluted to a final concentration of 2 *μ*M. UV–vis absorption and fluorescence spectra were recorded in different solvents (containing 0.01% DMSO) at 25ºC using a UV-vis spectrophotometer and a fluorescence spectrophotometer, respectively.

3.2. **Fluorescence quantum yield (*Φ_fl_*) determination**

According to previous report, fluorescence quantum yield determination of ***^Mp^BDP-CF_3_*** was measured using Rhodamine B as a reference with fluorescence quantum yield (*Φ_fl_*) of 0.97 in EtOH.^[2]^ The fluorescence quantum yield (*Φ_fl_*) was calculated by using the following formula:

$$\Phi_{fl}=\Phi_{fl,fluorescein}\left( \frac{F_{\mathrm{sample}}A_{\mathrm{fluorescein}}}{F_{\mathrm{fluorescein}}A_{\mathrm{sample}}} \right)\left( \frac{n_{\mathrm{sample}}^{2}}{n_{\mathrm{fluorescein}}^{2}} \right)$$

where n_sample_ and n_fluorescein_ are the refractive indices of the solvents used for the sample and fluorescein, respectively. F_sample_ and F_fluorescein_ are the integrated fluorescence emission of the sample and fluorescein, respectively. A_sample_ and A_fluorescein_ represent the absorbance of the sample and fluorescein at their respective excitation wavelengths. UV-visible absorption spectra were recorded on a UV2600 double-beam UV-Vis spectrophotometer (Shimadzu). Fluorescence spectra were recorded on an FL-7100 (Hitachi) spectrofluorometer. Measurements were carried out in 1 cm quartz cuvettes with a total sample volume of 3 mL.

3.3. **Theoretical calculation**

All DFT calculations were performed using the Gaussian 16 software package. The ground-state (S_0_) geometries of all species were optimized at the B3LYP/6-311G (d,p) level of theory. Vibrational frequency analyses were conducted to verify the nature of the stationary points, confirming minima by the absence of imaginary frequencies and transition states by the presence of a single imaginary frequency. Solvent effects of DMSO were incorporated using an appropriate solvation model.

## 3.4. Cell Culture

HepG2 cells, NIH 3T3 cells and LO2 cells were purchased from American Type Culture Collection (ATCC). The cells were cultured in DMEM or 1640 medium supplemented with 10% heatin-activated FBS, penicillin (100 U/mL), and streptomycin (100 *μ*g/mL) in a 37ºC incubator with 5% CO_2_. Before the experiment, the cells were precultured until confluence was reached to about 75%.

3.5. **Cell viability assay**

The cytotoxicity of ***^Mp^BDP-CF_3_*** was assessed using the CCK-8 assay. In this procedure, HepG2 cells and NIH 3T3 cells were initially seeded in 96-well plates at a density of approximately 5×10^3^ cells per well. After a 24 h incubation period, the medium was replaced with fresh medium containing ***^Mp^BDP-CF_3_*** at various concentrations. Following an additional 24 h incubation, the cells were washed with PBS, and a freshly prepared CCK-8 solution (100 *μ*L per well) in culture medium was added to each well. The cells were then incubated at 37°C for 1 h in the dark. Subsequently, absorbance was measured at 450 nm using a microplate reader (SpectraMax i3x, Molecular Devices).

3.6. **Cellular viscosity imaging**

Cells were subjected to treatment with Nystatin at varying concentrations (2 *µ*M and 20 *µ*M) or LPS (20 *µ*g/mL and 200 *µ*g/mL) for 0, 30, 60, and 120 min. Following this treatment, the cells were washed three times with PBS. Subsequently, 2 *µ*M of ***^Mp^BDP-CF_3_*** was introduced, and the cells were incubated for an additional 30 min, followed by three additional washes with PBS. Finally, fluorescence imaging was performed using an inverted fluorescence microscope.

3.7. **Cell polarity imaging**

Cells were subjected to treatment with DMSO at varying concentrations (5 *µ*L/mL and 10 *µ*L/mL) or DTT (2.5 mM and 5 mM) for 0, 10, 30, and 60 min. Following this treatment, the cells were washed three times with PBS. Subsequently, 2 *µ*M of ***^Mp^BDP-CF_3_*** was introduced, and the cells were incubated for an additional 30 min, followed by three additional washes with PBS. Finally, fluorescence imaging was performed using an inverted fluorescence microscope.

3.8. **NAFLD Imaging**

Cells were subjected to treatment with 500 *μ*M OA for 5, 10, and 15 h, respectively, and then stained with 2 *μ*M ***^Mp^BDP-CF_3_*** and BODIPY 493/503 for 30 min. Finally, fluorescence imaging was performed using an inverted fluorescence microscope.

## 4. Establishment of the NAFLD Nude Mouse Model

## All animal experiments were performed following the protocols evaluated and approved by the Experimental Animal Ethics Committee of Ningxia Medical University (Permit No. IACUC-2025331). Female BALB/c nude mice aged 6-8 weeks were purchased from Beijing Vital River Laboratory Animal Technology Co., Ltd. (Beijing, China).

A mouse NAFLD model was also established by feeding BALB/c mice (18 - 20 g) with a high-fat diet and dexamethasone (50 mg/kg, dissolved in ultrapure water, every other day for 14 days) intraperitoneal injection. The control mice were fed with normal feed for the same duration, following the literature methods.^[3]^ The model mice and normal mice were dissected after 14 days, and morphological observations were made on their main organs. Compared with that of normal mice, the liver color of NAFLD model mice was significantly lighter, appearing pale yellow. The liver volume increased significantly, with blunt edges, and an oily texture could be observed on the surface. BALB/c nude mice (control group, *n* = 3) and NAFLD BALB/c nude mice (experimental group, *n* = 3) were intravenously injected with ***^Mp^BDP-CF_3_*** (5 mg/kg, 200 *μ*L) via the tail vein. In vivo fluorescence imaging was performed at different time points (0 - 45 min) post-injection. After imaging, the mice were euthanized, and the major organs were harvested for ex vivo fluorescence imaging.

## 5. Supplementary Figures

**Table S1.** Comparison of previously reported lipid droplets-targeting fluorescent probes for NAFLD.

**Table S2.** Photophysical data for ***^Mp^BDP-CF_3_*_._**

**Figure S1.** Gaussian calculation and simulation of the UV-vis absorption and fluorescence emission spectra of a) ***^Mp^BDP-CF_3_*** and b) ^Me^BDP-CF_3_.

**Figure S2.** Scatter plot and visualization of interaction region index in ^Me^BDP-CF_3_.

**Figure S3.** UV−vis absorption spectra were recorded for 2.0 *μ*M solutions of a) ***^Mp^BDP-CF_3_*** and c) ^Me^BDP-CF_3_ in various solvents. Fluorescence emission spectra were similarly obtained for 2.0 *μ*M solutions of b) ***^Mp^BDP-CF_3_*** and d) ^Me^BDP-CF_3_ across different solvents.

**Figure S4.** a) UV−vis absorption spectra and b) fluorescence emission spectra of ***^Mp^BDP-CF_3_*** (2.0 *μ*M) in 1,4-dioxane/PBS mixtures of various fractions (60-100%). c) Linear fitting of ***^Mp^BDP-CF_3_*** in 1,4-dioxane/PBS (*n* = 3).

**Figure S5.** Optical photographs recorded under UV (365 nm) for ***^Mp^BDP-CF_3_*** in a) glycerol/PBS and b) 1,4-dioxane/PBS mixtures of various fractions (0−100%).

**Figure S6.** a) Evaluation of the cytotoxicity of ***^Mp^BDP-CF_3_*** in various cell lines determined by cell viability assay. Error bars represent mean ± SD (*n* = 4).

**Figure S7.** a) HepG2 cells were pretreated with LPS (20 *μ*g/mL) and Nystatin (2.0 *μ*M/mL) for different durations (0, 30, 60, and 120 min), followed by incubation with 2.0 *μ*M ***^Mp^BDP-CF_3_*** for 30 min before fluorescence imaging. Scale bar: 50 *μ*m. b) Quantitative analysis of the mean fluorescence intensity corresponding to the cellular images displayed in figure S7a (*n* = 3). *One-way ANOVA, mean ± SD, *P < 0.05, ****P < 0.0001, ns represents no significance.*

**Figure S8.** a) HepG2 cells were pretreated with DMSO (5 *μ*L/mL) and DTT (2.5 mM) for different durations (0, 10, 30 and 60 min), followed by incubation with 2.0 *μ*M ***^Mp^BDP-CF_3_*** for 30 min before fluorescence imaging. Scale bar: 50 *μ*m. b) Quantitative analysis of the mean fluorescence intensity corresponding to the cellular images displayed in figure S8a (*n* = 3). *One-way ANOVA, mean ± SD, ****P < 0.0001, ns represents no significance.*

**Figure S9.** a) LO2 cells were pretreated with 500 *μ*M OA for 5, and 10 h, respectively, followed by incubation with a staining solution containing 2.0 *μ*M BODIPY 493/503 and 2.0 *μ*M ***^Mp^BDP-CF_3_*** for 30 min prior to fluorescence imaging. Scale bar: 50 *μ*m. b) Quantitative analysis of the average fluorescence intensities in the green and red channels is presented in figure S9a (*n* = 3). *One-way ANOVA, mean ± SD, ****P < 0.0001, ns represents no significance.*

6. **NMR and HRMS spectra**

**Figure S10.** ^1^H NMR spectrum (400 MHz) of ***Compound 1*** in CDCl_3_ at 25°C.

**Figure S11.** ^13^C NMR spectrum (100 MHz) of ***Compound 1*** in CDCl_3_ at 25°C.

**Figure S1****2.** ^1^H NMR spectrum (400 MHz) of ***Compound 2*** in CDCl_3_ at 25°C.

**Figure S13.** ^13^C NMR spectrum (100 MHz) of ***Compound 2*** in CDCl_3_ at 25°C.

**Figure S14.** ^1^HNMR spectrum (400 MHz) of ***^Mp^BDP-CF_3_*** in CDCl_3_ at 25°C.

**Figure S15.** ^13^CNMR spectrum (100 MHz) of ***^Mp^BDP-CF_3_*** in CDCl_3_ at 25°C.

**Figure S16.** HRMS of ***^Mp^BDP-CF_3_***.

7. **References**
[1] S. Kim, J. Bouffard, Y. Kim, *Chem. Eur. J.* **2015**, *21*, 17459.
[2] S. Kenmoku, Y. Urano, H. Kojima, T. Nagano, *J. Am. Chem. Soc.* **2007**, *129*, 7313.
[3] Y. Ma, B. Guo, J. Ge, L. Chen, N. Lv, X. Wu, J. Chen, Z. Chen, *Anal. Chem.* **2022**, *94*, 12383.
[4] H. Huang, Y. Bu, Z. Yu, M. Rong, R. Li, Z. Wang, J. Zhang, X. Zhu, L. Wang, H. Zhou, *Anal. Chem.* **2022**, *94*, 13396.
[5] Y. Liu, F. Liu, T. Wu, F. Yuan, Z. An, M. Sun, J. Jing, X. Zhang, *Chem.-Asian J.* **2026**, *21*, e00953.
[6] Y. Yang, R. Guo, K. Hu, M. Xu, T. Liang, W. Lin, *Luminescence* **2024**, *39*, e4749.
[7] J. Sha, W. Liu, X. Zheng, Y. Guo, X. Li, H. Ren, Y. Qin, J. Wu, W. Zhang, C. Lee, P. Wang, *Anal. Chem.* **2023**, *95*, 15350.
[8] H. W. Lee, I. Lee, S. Lee, Y. R. Kim, H. M. Kim, *ACS Sens.* **2022**, *7*, 1027.
[9] M. Shimanuki, R. Uda, S. Shiozaki, T. Yoshihara, *ACS Appl. Bio Mater.* **2025**, *8*, 6193.
[10] C. Xiang, X. Wang, R. Zheng, X. Liu, X. Han, Z. Gui, L. Hu, G. Niu, *Chem. Biomed. Imaging* **2026**, *4*, 638.
[11] X. Tan, Y. Liu, S. Jiang, Z. Zheng, G. Feng, *Chem. Biomed. Imaging* **2026**, *4*, 924-933.
[12] Y. Wang, K. Yu, J. Yang, J. Wang, Z. Wang, A. Sun, C. Wang, L. Hu, H. Wang, *Chem. Biomed. Imaging* **2026**, *4*, 823-836.
[13] J. Hao, S. Shi, Y. Zhang, X. Li, X. Ren, J. Gao, S. Liu, H. Zhang, J. Wu, B. Zhang, *Sens. Actuators, B.* **2025**, *433*, 137581.
